# White Matter Correlates of Neuropsychological Dysfunction in Systemic Lupus Erythematosus

**DOI:** 10.1371/journal.pone.0028373

**Published:** 2012-01-26

**Authors:** Rex E. Jung, Robert S. Chavez, Ranee A. Flores, Clifford Qualls, Wilmer L. Sibbitt, Carlos A. Roldan

**Affiliations:** 1 Department of Neurosurgery, University of New Mexico, Albuquerque, New Mexico, United States of America; 2 Mind Research Network, University of New Mexico, Albuquerque, New Mexico, United States of America; 3 Department of Mathematics, and University of New Mexico, Albuquerque, New Mexico, United States of America; 4 Department of Internal Medicine, University of New Mexico, Albuquerque, New Mexico, United States of America; Queensland Institute of Medical Research, Australia

## Abstract

Patients diagnosed with Systemic Lupus Erythematosus have similar levels of neuropsychological dysfunction (i.e., 20–50%) as those with Neuropsychiatric Systemic Lupus Erythematosus (NPSLE). We hypothesized a gradient between cognition and white matter integrity, such that strongest brain-behavior relationships would emerge in NPSLE, intermediate in non-NPSLE, and minimal in controls. We studied thirty-one patients (16 non-NPSLE; 15 NPSLE), ranging in age from 18 to 59 years old (100% female), and eighteen age and gender matched healthy controls. DTI examinations were performed on a 1.5T scanner. A broad neuropsychological battery was administered, tapping attention, memory, processing speed, and executive functioning. The Total z-score consisted of the combined sum of all neuropsychological measures. In control subjects, we found no significant FA-Total z-score correlations. NPSLE, non-NPSLE, and control subjects differed significantly in terms of Total z-score (NPSLE = −2.25+/−1.77, non-NPSLE = −1.22+/−1.03, Controls = −0.10+/−.57; F = 13.2, p<.001). In non-NPSLE subjects, FA within the right external capsule was significantly correlated with Total z-score. In NPSLE subjects, the largest FA-Total z-score clusters were observed within the left anterior thalamic radiation and right superior longitudinal fasciculus. In subsequent analyses the largest number of significant voxels linked FA with the Processing Speed z-score in NPSLE. The current results reflect objective white matter correlates of neuropsychological dysfunction in both NPSLE and (to a lesser degree) in non-NPSLE. non-NPSLE and NPSLE subjects did not differ significantly in terms of depression, as measured by the GDI; thus, previous hypotheses suggesting moderating effects of depression upon neuropsychological performance do not impact the current FA results.

## Introduction

Cognitive dysfunction is a significant (i.e., 20–42%) comorbid characteristic of systemic lupus erythematosus [1]. However, SLE patients with significant neurological involvement – collectively known as neuropsychiatric systemic lupus erythematosus (NPSLE) – have comparable levels of cognitive dysfunction (27–52%) [2]. NPSLE has been hypothesized to have multiple causal etiologies, many of which are hypothesized to affect white matter functioning, including: inflammatory, hypoperfusion, thrombotic, vasculitic, atherosclerotic, and/or cardioembolic, further exacerbated by antibody, cytokine, and cytotoxic mediators [3], [4], [5], [6].

Few prospective studies have linked cognitive decline to white matter changes across NPSLE and SLE patient cohorts. In SLE subjects (here forward “non NPSLE”), subtle correlations between neuropsychological measures and white matter integrity, measured by diffusion tensor imaging (DTI), have been observed [7]. The current research is conducted in the identical cohort reported on previously [8], and is therefore *post hoc*.” Based on previous research [8], [9], [10], [11], [12], we anticipate a gradient between cognition and white matter integrity, such that strongest brain-behavior relationships would emerge in NPSLE, intermediate in non-NPSLE, and minimal in controls.

## Methods

### Sample

Written informed consent was obtained from all subjects prior to participation in the experimental protocol. The study was approved by the institutional review board of the University of New Mexico, and consistent with the Declaration of Helsinki.

Thirty-one female patients were recruited from the Rheumatology Clinics of the University of New Mexico, and ranged in age from 18 to 59 years old. All subjects were diagnosed with lupus based on the 1997 American College of Rheumatology Revised Criteria. The SLE Disease Activity Index (SLEDAI) was used to assess current disease activity [13]. The Systemic Lupus International Collaborative Clinics (SLICC) Damage Index was used to assess accumulated damage due to various organs [14]. Patients with SLE were enrolled prospectively as encountered in either University outpatient or inpatient settings, without regard of SLE disease activity and severity. No patients were scanned or tested in the presence of active neuropsychiatric symptoms (e.g., acute confusional state, psychosis), although most had some level of chronic cognitive dysfunction. No non-NPSLE patient complained of cognitive dysfunction, thus they remained categorized under ACR guidelines as SLE. All assessments (clinical, behavioral, imaging) were conducted within one month following onset of neuropsychiatric events.

Sixteen patients met the American College of Rheumatology case definitions for Neuropsychiatric (NP) events in SLE, defined as acute stroke or transient ischemic attack (TIA), acute confusional state, moderate cognitive dysfunction, seizures, or psychosis [15]. Fifteen patients had non-NPSLE, but met criteria for Systemic Lupus Erythematosus. Patients were compared to eighteen female, age matched, healthy controls. The SLE Disease Activity Index (SLEDAI) was used to assess current disease activity across nine organ systems including: central nervous system, vascular, renal, musculoskeletal, serosal, dermal, immunologic, constitutional, and hematologic [13]. Forty-nine (of fifty-three) subjects were reported in a previous study showing DTI differences between NPSLE and non-NPSLE patients [8]; therefore the design is *post hoc*. The current sample differs from our previous report in that we focus exclusively on female subjects (previous sample included one male NPSLE patient, one male non NPSLE patient, and two male controls). We encourage readers to review that open access paper for additional information regarding clinical characteristics of patients, lesion load, and statistical comparisons referred to within the current study (http://www.biomedcentral.com/1471-2377/10/65).

### Image Acquisition

MR examinations were conducted on a 1.5T scanner using an 8-channel phased array head coil. DTI was acquired as follows: [TE = 92 ms, TR = 10000 ms; FOV = 256 mm; 64 slices; 2 mm slice thickness; 12 diffusion directions; Total acquisition time = 4∶32]. DTI was performed twice and averaged to increase signal to noise ratio.

### Behavioral Measure

The neuropsychological battery is in general compliance with the recommendations of the American College of Rheumatology [16]. The Wide Range Achievement Test–3 (WRAT-3) Reading subtest, was used as a measure of premorbid intellectual functioning. Depressive symptoms were assessed with the Geriatric Depression Inventory (GDI). The following tests comprised z-score domains: 1) Trail Making Test Part A, Digit Span–Total Score = Attention z-score; 2) Hopkins Verbal Learning Test–Total Learning Score, Delayed Recall Score = Verbal Memory z-score; 3) Rey Complex Figure Test–Immediate Recall, Delay Recall Scores = Visual Memory z-score; 4) Trail Making Test Part B, Controlled Oral Word Association Test–Total Score, Wisconsin Card Sort–Perseverative Error Score = Executive z-score; 5) Digit Symbol, Symbol Search scores from the Wechsler Adult Intelligence Scale-3 = Processing Speed z-score [17]. The Total z-score consisted of the combined sum of all neuropsychological measures. All subjects individual scores were compared to age matched normative control samples before being summated to z-scores for statistical comparison [18].

### Radiological Reads

Brain atrophy and lesions were quantified by one of the authors (WLS), blind to group status of individual scan (NPSLE, non NPSLE, control). Methodology by which lesions loads are quantified in NPSLE cohorts have been described previously by our group [4], [19], [20]. Briefly, MRI scans were classified as “normal” (i.e., no focal abnormalities or diffuse lesions), “abnormal” (i.e., any focal or diffuse abnormality on imaging), or “small focal lesion” (i.e., less than 3 mm in diameter on imaging). Similarly, distinctions were made between “old infarcts” (i.e., hyperintense lesions greater than 3 mm associated with local encephalomalacia and typical changes on T1 images), or “recent infarcts” (i.e., hyperintense lesions associated with restricted diffusion, but without local encephalomalacia. Finally, lesions were localized to subcortical white matter, periventricular white matter, or deep white matter. Fisher's exact test was used to determine statistical group differences between NPSLE and non NPSLE patients across radiological measures (Table 1).

**Table 1:** 

	Controls	NPSLE	non-NPSLE	F	p
**Demographics, IQ, & Mood**					
*Age*	32.2	38.1	37.5	1.31	0.28
*Premorbid IQ (WRAT-3)*	47	44.9	46	0.40	0.67
*GDI (Depression)*	3.9	12.9	8.9	9.30	**0.0004**
*Total z-score*	−0.1	−2.25	−1.22	13.20	**0.001**
				**Fisher's Exact**	
**Medications**					
*Prednisone*		15	13	0.33	
*Cyclophosphamide*		6	4	0.46	
*Mycophenalate*		2	2	1.00	
*Retuximab*		0	1	1.00	
*Hydroxychloroquine*		8	8	1.00	
*Aspirin*		7	2	0.06	
*Methotrexate*		0	4	0.10	
*Coumadin*		6	2	0.12	
*Clopidrel*		1	0	0.48	
*SSRI*		4	3	0.69	
*Anti Seizure*		2	1	0.60	
*Narcotics*		4	0	**0.04**	
*Depressants*		2	0	0.23	
*Pulse Chemotherapy*		4	2	0.40	
*Other*		0	1	1.00	
**Radiological Findings**					
*Cortical Atrophy*		3	0	0.23	
*Subcortical White Matter Lesions*		4	10	0.07	
*Periventricular White Matter Lesions*		10	4	0.09	
*Deep White Matter Lesions*		5	2	0.20	
*Old Infarction*		5	1	**0.02**	
*Recent Infarction*		1	0	1.00	

### Analysis

Tract-Based Spatial Statistics (TBSS) allows measurement of water movement along white matter tracts on a voxel-by-voxel basis across the entire brain [21]. Detailed methods by which we obtained significant FA differences between NPSLE, non-NPSLE, and control subjects are described in our previous paper [8]. For the current *post hoc* analysis, the fractional anisotropy (FA) image of each subject was normalized to a 1×1×1 mm^3^ FA template in the Montreal Neurological Institute (MNI) space using the non-linear registration algorithm FNIRT/FSL (www.fmrib.ox.ac.uk/fsl/). We first correlated the Total z score with FA across each subject group (e.g., NPSLE, non NPSLE, controls) using FSL's General Linear Model (GLM) tool (www.fmrib.ox.ac.uk/fsl/fsl/list). Age was entered into the model as a nuisance variable. All results are corrected at p-values<.05 after controlling for family wise error rate. If significant, then subdomain z-scores (e.g., Processing Speed z score) were then correlated with FA for each subject group. The correlation inferences were tested using permutation methods with FSL's Randomise (www.fmrib.ox.ac.uk/fsl/randomise). We ran 5000 two-tailed Monte Carlo permutation tests, (i.e., both positive and negative associations) for each of the correlations between neuropsychological composite measures and FA. [22], [23]

## Results

### Demographic data

NPSLE, non-NPSLE, and control subjects did not differ significantly in terms of age (NPSLE = 38.1, non-NPSLE = 37.5, Controls = 32.2; F = 1.31, p = .28), and premorbid intellectual functioning (NPSLE = 44.9+/−7.1; non-NPSLE = 46.0+/−8.0; controls = 47.0+/−7.09; F = .40, p = .67). Control subjects differed from both non-NPSLE (t = 2.44, p = .05) and NPSLE (t = 4.28, p = .0003) on the GDI, a measure of depressive symptomatology (NPSLE = 12.9+/−6.1; non-NPSLE = 8.9+/−7.1; controls = 3.9+/−4.8; F = 9.3, p = .0004); however, NPSLE and non-NPSLE scores on the GDI were not significantly different (t = 1.89, p = .12).

### Neuropsychological data

One way ANOVA revealed that NPSLE, non-NPSLE, and control subjects differed significantly in terms of Total z-score (NPSLE = −2.25+/−1.77, non-NPSLE = −1.22+/−1.03, Controls = −0.10+/−.57; F = 13.2, p<.001), with *post hoc* analysis indicating significant NPSLE-non-NPSLE differences on Total z-score (Tukey HSD; Mean difference = 1.03, Standard Error = .42, Significance = .045). The Total z-score represents roughly 1 standard deviation decline (from premorbid estimates) in overall cognitive functioning for the non-NPSLE group, and a 2 standard deviation decline in functioning for the NPSLE group. Only one subdomain z-score (Processing Speed) showed significant NPSLE-non-NPSLE differences (Tukey HSD; Mean difference = 1.47, Standard Error = .47, Significance = .008).

### Clinical, laboratory and treatment features

Table 1 summarizes demographic, clinical, medication, and radiological features between NPSLE and non-NPSLE patients. The patient groups differed significantly on measures of disease activity (SLEDAI-Total: t = 2.88, p = .009) and accumulated organ damage (SLICC-Total: t = 3.44, p = .002). However, when neurological components were removed from these measures, NPSLE patients did not differ from non-NPSLE on the SLEDAI (t = 1.04, p = .31); they did differ on the SLICC (t = 2.17, p = .044), although no individual subscores were significantly different between patient groups. Patient groups did not differ in terms of prevalence of any medications being taken, except for narcotics (p = .04), including average mg of prednisone taken per day (NPSLE = 9.69; non-NPSLE = 5.23; t = 1.58, p = .13). Finally, patient groups did not differ in terms of radiological readings, except for evidence of old infarction (p = .02).

### FA – Neuropsychological Correlations by group

**Figure 1:**
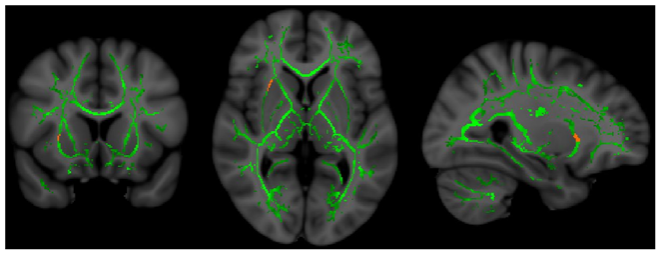
FA-Total z-score relationships in non-NPSLE. Significant regions (red/yellow) in which non-NPSLE patients had significant correlations between Fractional Anisotropy and Total z-score. Left – coronal view (front to back of head); middle – axial view (top to bottom of head); right – sagittal view (side to side of head). Green represents the center of major white matter tracts which represent the total regions of statistical analyses.

**Figure 2:**
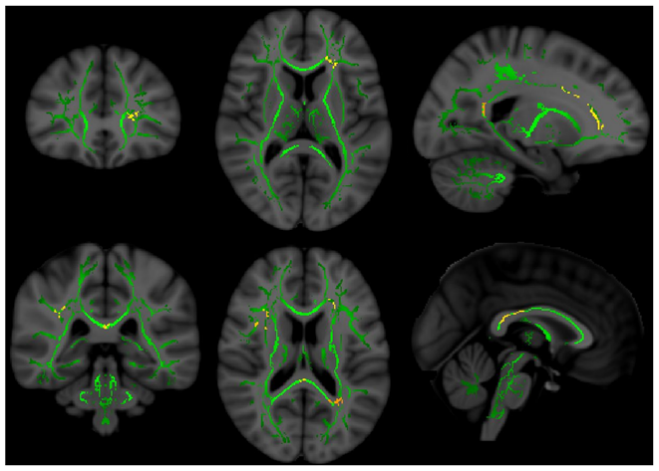
FA-Total z-score relationships in NPSLE. Significant regions (red/yellow) in which NPSLE patients had significant correlations between FA and Total z-score. Left – coronal view (front to back of head); middle – axial view (top to bottom of head); right – sagittal view (side to side of head). Green represents the center of major white matter tracts which represent the total regions of statistical analyses.

**Figure 3:**
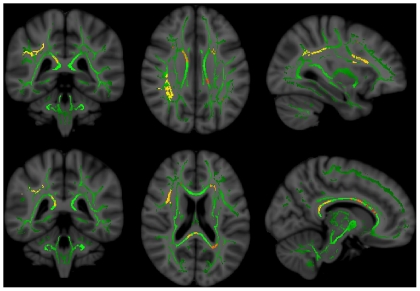
FA-Processing Speed z-score in NPSLE. Significant regions (red/yellow) in which NPSLE patients had significant correlations between FA and Processing Speed z-score. Left – coronal view (front to back of head); middle – axial view (top to bottom of head); right – sagittal view (side to side of head). Green represents the center of major white matter tracts which represent the total regions of statistical analyses.

In control subjects, we found no significant regions in which FA was correlated with Total z-score. In non-NPSLE subjects, FA within the right external capsule was significantly correlated with Total z-score (p = .029)(Figure 1). No subcomponents of the Total z-score were significantly correlated with FA in non-NPSLE. In NPSLE subjects, FA within numerous regions of the white matter were correlated with Total z-score, the largest cluster being within the left anterior thalamic radiation (p = .004) and right superior longitudinal fasciculus (.007<p<.012)(Figure 2). In subsequent analyses, all subcomponent domains of the neuropsychological battery were related to FA in NPSLE (all p<0.05) (Table 2), with the largest number of voxels (i.e., 2645) linking FA and the Processing Speed z-score (Figure 3).

**Table 2:** 

Voxels	p-values	MNIx	MNIy	MNIz	Approximate Tract
**Total z-score**					
499	0.004	−20	33	1	Left Anterior Thalamic Radiation
391	0.006	4	−37	16	Splenium Corpus Callosum
331	0.007	36	−44	22	Right Superior Longitudinal Fasciculus
249	0.012	39	10	16	Right Superior Longitudinal Fasciculus
175	0.02	−19	−52	11	Left arm of Forceps Major
170	0.022	2	6	23	Mid-body Corpus Callosum
136	0.031	31	12	30	Right Anterior Corona Radiata
105	0.049	19	26	19	Right arm of Forceps Minor
Total = 2056					
**Attention z-score**					
412	0.007	17	−42	9	Splenium Corpus Callosum
271	0.014	1	8	22	Mid-body Corpus Callosum
253	0.016	39	6	17	Right Superior Longitudinal Fasciculus
131	0.049	−27	−55	15	Left arm of Forceps Major
Total = 1067					
**Verbal Memory z-score**					
113	0.031	−31	−52	13	Left Inferior Longitudinal Fasciculus
Total = 113					
**Visual Memory z-score**					
1156	0.001	−33	−58	10	Left IF-OF
216	0.02	9	21	15	Right arm of Forceps Minor
177	0.027	−35	−50	16	Left Superior Longitudinal Fasciculus
147	0.038	−47	−3	20	Left Superior Longitudinal Fasciculus
Total = 1696					
**Executive z-score**					
369	0.014	43	3	16	Right Superior Longitudinal Fasciculus
298	0.018	−22	28	13	Left Anterior Thalamic Radiation
163	0.04	36	−38	23	Right Superior Longitudinal Fasciculus
Total = 830					
**Processing Speed z-score**					
585	0.005	41	−40	23	Right Superior Longitudinal Fasciculus
344	0.009	12	−41	13	Splenium Corpus Callosum
308	0.01	43	6	12	Right Superior Longitudinal Fasciculus
220	0.019	−24	22	17	Left Anterior Thalamic Radiation
192	0.025	2	4	24	Mid-body Corpus Callosum
180	0.028	−19	−52	14	Left arm of Forceps Major
149	0.037	−15	−33	29	Splenium Corpus Callosum
145	0.038	29	12	30	Right Anterior Corona Radiata
143	0.038	−36	−51	14	Left Superior Longitudinal Fasciculus
132	0.042	19	26	19	Right arm of Forceps Minor
127	0.044	−26	4	31	Left Superior Corona Radiata
120	0.046	13	29	8	Genu Corpus Callosum
Total = 2645	*Total voxels in skeleton≈77,000 (Smith et al., 2006, NeuroImage)*				

## Discussion

This is the first study to evaluate neuropsychological and white matter functioning in a well-matched cohort of female NPSLE, non-NPSLE, and control subjects. Importantly, non-NPSLE and NPSLE subjects did not differ significantly in terms of depression, as measured by the GDI; thus, previous hypotheses suggesting moderating effects of depression upon neuropsychological performance [2], [24] do not appear to impact the current FA results. Similarly, FA differences could not be explained by non-neurological group differences in disease activity (SLEDAI), accumulation of disease burden (SLICC), or medication effects. We know of no evidence linking the higher incidence of narcotic medication use to FA abnormalities. Finally, our results correspond well with the first article showing FA differences between non-NPSLE and NPSLE subjects [11]. Not only do our results replicate similar regions of abnormality in the current cohort of NPSLE patients, including thalamus, corpus callosum, and fronto-parietal white matter regions, but link these regional abnormalities to specific neurocognitive deficits.

These finding represent preliminary evidence that the neurocognitive effects of NPSLE differ significantly from non-NPSLE even when tightly matched by age, sex, medication use, mood symptoms, and non-neurological disease activity and accumulated disease burden. Moreover, these neurocognitive effects are well correlated with FA changes within white matter regions outside of overt lesions [8]. These FA changes are consistent with postmortem studies showing subtle findings in fatal NPSLE including global ischemic changes, edema, microhemorrhages, glial hyperplasia, diffuse neuronal/axonal loss, and microthromboemboli [4]. The strongest relationship between white matter integrity and neuropsychological domain was observed for the Processing Speed z-score, a measure sensitive to global white matter functioning. This result partially replicates and extends findings from our laboratory [25] and others [7] showing relationships between measures sensitive to white matter functioning and measures of white matter integrity.

Neuroimaging of complex cognition has evolved beyond strict localization of function [26] to greater appreciation of a network approach implicating rapid and accurate transfer of information between coordinated brain regions [27]. Lesion or degradation of white matter throughout this network is associated with decline in cognitive functioning across a wide range of tasks [28]. While several of our NPSLE patients experienced acute, local infarction in the past, many of the white matter tracts implicated in the current study, (e.g., superior longitudinal fasciculus, corpus callosum, anterior thalamic radiation) connect both subcortical and cortical association cortices in service of complex neuropsychological performance [29]. Thus, we interpret the current findings as reflecting reorganization of residual cognitive functioning in relatively intact white matter subserving distributed cognitive networks.

There are limitations to this approach, including the fact that five of the NPSLE patients had overt lesions due to stroke, which in turn could have led to Wallerian degeneration at least partially responsible for diffuse white matter changes [30]. It is important to distinguish the current findings, which reflect FA-neuropsychological relationships, likely reflecting brain integrity, as opposed to our previous findings in the same cohort wherein FA differences reflected white matter damage. Future research will be necessary to disentangle the complex interaction between white matter lesions, subsequent Wallerian degeneration, and compensatory changes within networks subserving cognitive performance within experimental cohorts comprised of NPSLE and non-NPSLE patients. Future studies with large clinical samples amenable to subgroup comparisons (e.g., with/without stroke) will assist in determining the additional impact of these variables upon neuropsychological functioning and white matter status. Strengths of the current study include: 1) a relatively young cohort, 2) comparison of NPSLE, non-NPSLE, and control cohorts, and 3) simultaneous administration of cognitive, mood, and white matter measures. In summary, the current results reflect objective white matter correlates of neuropsychological dysfunction in well matched samples of female NPSLE and non-NPSLE patients.

## References

[1] Carbotte RM, Denburg SD, Denburg JA (1986). Prevalence of cognitive impairment in systemic lupus erythematosus.. J Nerv Ment Dis.

[2] Monastero R, Bettini P, Del Zotto E, Cottini E, Tincani A (2001). Prevalence and pattern of cognitive impairment in systemic lupus erythematosus patients with and without overt neuropsychiatric manifestations.. J Neurol Sci.

[3] Sabet A, Sibbitt WL, Stidley CA, Danska J, Brooks WM (1998). Neurometabolite markers of cerebral injury in the antiphospholipid antibody syndrome of systemic lupus erythematosus.. Stroke.

[4] Sibbitt WL, Brooks WM, Kornfeld M, Hart BL, Bankhurst AD (2010). Magnetic resonance imaging and brain histopathology in neuropsychiatric systemic lupus erythematosus.. Semin Arthritis Rheum.

[5] Navarrete MG, Brey RL (2000). Neuropsychiatric Systemic Lupus Erythematosus.. Curr Treat Options Neurol.

[6] Luyendijk J, Steens SC, Ouwendijk WJ, Steup-Beekman GM, Bollen EL (2011). Neuropsychiatric systemic lupus erythematosus: lessons learned from magnetic resonance imaging.. Arthritis Rheum.

[7] Filley CM, Kozora E, Brown MS, Miller DE, West SG (2009). White matter microstructure and cognition in non-neuropsychiatric systemic lupus erythematosus.. Cogn Behav Neurol.

[8] Jung RE, Caprihan A, Chavez RS, Flores RA, Sharrar J (2010). Diffusion tensor imaging in neuropsychiatric systemic lupus erythematosus.. BMC Neurol.

[9] Emmer BJ, Veer IM, Steup-Beekman GM, Huizinga TW, van der Grond J (2010). Tract-based spatial statistics on diffusion tensor imaging in systemic lupus erythematosus reveals localized involvement of white matter tracts.. Arthritis Rheum.

[10] Bosma GP, Huizinga TW, Mooijaart SP, Van Buchem MA (2003). Abnormal brain diffusivity in patients with neuropsychiatric systemic lupus erythematosus.. AJNR Am J Neuroradiol.

[11] Hughes M, Sundgren PC, Fan X, Foerster B, Nan B (2007). Diffusion tensor imaging in patients with acute onset of neuropsychiatric systemic lupus erythematosus: a prospective study of apparent diffusion coefficient, fractional anisotropy values, and eigenvalues in different regions of the brain.. Acta Radiol.

[12] Zhang L, Harrison M, Heier LA, Zimmerman RD, Ravdin L (2007). Diffusion changes in patients with systemic lupus erythematosus.. Magn Reson Imaging.

[13] Bombardier C, Gladman DD, Urowitz MB, Caron D, Chang CH (1992). Derivation of the SLEDAI. A disease activity index for lupus patients. The Committee on Prognosis Studies in SLE.. Arthritis Rheum.

[14] Gladman DD, Urowitz MB, Goldsmith CH, Fortin P, Ginzler E (1997). The reliability of the Systemic Lupus International Collaborating Clinics/American College of Rheumatology Damage Index in patients with systemic lupus erythematosus.. Arthritis Rheum.

[15] (1999). The American College of Rheumatology nomenclature and case definitions for neuropsychiatric lupus syndromes.. Arthritis Rheum.

[16] Kozora E, Ellison MC, West S (2004). Reliability and validity of the proposed American College of Rheumatology neuropsychological battery for systemic lupus erythematosus.. Arthritis Rheum.

[17] Lezak MD, Howieson DB, Loring DW, Hannay HJ, Fischer JS (2004). Neuropsychological Assessment.

[18] Strauss E, Sherman EMS, Spreen O (2006). A Compendium of Neuropsychological Tests: Administration, Norms, and Commentary.

[19] Sibbitt WL, Haseler LJ, Griffey RH, Hart BL, Sibbitt RR (1994). Analysis of cerebral structural changes in systemic lupus erythematosus by proton MR spectroscopy.. AJNR Am J Neuroradiol.

[20] Sibbitt WL, Schmidt PJ, Hart BL, Brooks WM (2003). Fluid Attenuated Inversion Recovery (FLAIR) imaging in neuropsychiatric systemic lupus erythematosus.. J Rheumatol.

[21] Smith SM, Jenkinson M, Johansen-Berg H, Rueckert D, Nichols TE (2006). Tract-based spatial statistics: voxelwise analysis of multi-subject diffusion data.. Neuroimage.

[22] Smith SM, Jenkinson M, Woolrich MW, Beckmann CF, Behrens TE (2004). Advances in functional and structural MR image analysis and implementation as FSL.. Neuroimage.

[23] Woolrich MW, Jbabdi S, Patenaude B, Chappell M, Makni S (2009). Bayesian analysis of neuroimaging data in FSL.. Neuroimage.

[24] Kozora E, Ellison MC, West S (2006). Depression, fatigue, and pain in systemic lupus erythematosus (SLE): relationship to the American College of Rheumatology SLE neuropsychological battery.. Arthritis Rheum.

[25] Brooks WM, Jung RE, Ford CC, Greinel EJ, Sibbitt WL (1999). Relationship between neurometabolite derangement and neurocognitive dysfunction in systemic lupus erythematosus.. J Rheumatol.

[26] Broca MP (1861). Remarques sur le siege de la faculte du langage articule suivies d'une observation d'aphemie.. Bulletin de la Societe Anatomique Paris.

[27] Cabeza R, Nyberg L (2000). Imaging cognition II: An empirical review of 275 PET and fMRI studies.. J Cogn Neurosci.

[28] Filley CM (2001). The behavioral neurology of white matter.

[29] Schmahmann JD, Smith EE, Eichler FS, Filley CM (2008). Cerebral white matter: neuroanatomy, clinical neurology, and neurobehavioral correlates.. Ann N Y Acad Sci.

[30] Thomalla G, Glauche V, Koch MA, Beaulieu C, Weiller C (2004). Diffusion tensor imaging detects early Wallerian degeneration of the pyramidal tract after ischemic stroke.. Neuroimage.

